# Rhodamine-Based Cyclic Hydroxamate as Fluorescent pH Probe for Imaging of Lysosomes

**DOI:** 10.3390/ijms242015073

**Published:** 2023-10-11

**Authors:** Young Ju Kim, Mina Jang, Jongtae Roh, Yoon Jeong Lee, Hee Jung Moon, Jimin Byun, Jihyun Wi, Sung-Kyun Ko, Jinsung Tae

**Affiliations:** 1 Department of Chemistry, Yonsei University, Seoul 03722, Republic of Korea; excel1903@hanmail.net (Y.J.K.); leeyj950513@gmail.com (Y.J.L.); bluechu@gmail.com (H.J.M.); minnye3@naver.com (J.B.); j-hyunii83@hanmail.net (J.W.); 2 Chemical Biology Research Center, Korea Research Institute of Bioscience and Biotechnology (KRIBB), Cheongju 28116, Republic of Korea; jangmina@kmedihub.re.kr (M.J.); jtroh@kribb.re.kr (J.R.); 3 New Drug Development Center, Daegu-Gyeongbuk Medical Innovation Foundation, Daegu 41061, Republic of Korea; 4 KRIBB School of Bioscience, Korea University of Science and Technology (UST), Daejeon 34141, Republic of Korea

**Keywords:** fluorescent probe, rhodamine B, hydroxamate, fluorescent imaging, acidic pH

## Abstract

Monitoring the microenvironment within specific cellular regions is crucial for a comprehensive understanding of life events. Fluorescent probes working in different ranges of pH regions have been developed for the local imaging of different pH environments. Especially, rhodamine-based fluorescent pH probes have been of great interest due to their ON/OFF fluorescence depending on the spirolactam ring’s opening/closure. By introducing the *N*-alkyl-hydroxamic acid instead of the alkyl amines in the spirolactam of rhodamine, we were able to tune the pH range where the ring opening and closing of the spirolactam occurs. This six-membered cyclic hydroxamate spirolactam ring of rhodamine B proved to be highly fluorescent in acidic pH environments. In addition, we could monitor pH changes of lysosomes in live cells and zebrafish.

## 1. Introduction

Intracellular pH levels are highly important in controlling various biological processes, including cell proliferation, apoptosis, metabolism, and signal transduction [1,2,3,4]. Specific pH ranges in organelles, such as lysosomal pH (4.5–5.5), cytosolic pH (7.0–7.4) and mitochondrial pH (7.2–8.0), are required to maintain cellular functions [5]. Especially, lysosomes, membrane-bound cell organelles containing acid hydrolase enzymes capable of digesting macromolecules, require a low pH condition (4.5–5.5) for their optimal actions [6]. Lysosomal dysfunctions may induce abnormal protein accumulation, causing neurodegenerative diseases (such as Alzheimer’s disease and Parkinson’s disease) and lysosomal storage diseases (such as Gaucher disease and Niemann–Pick type C disease) [7]. Accordingly, the monitoring of pH values can provide critical information for studying biological and pathological processes leading to these diseases [8].

Several pH measurement methods, such as microelectrodes, NMR, absorbance spectroscopy and fluorescence spectroscopy, have been developed to observe the pH changes inside of cells. Above all, the fluorescence technique using pH sensitive molecular probes has been developed extensively due to its several advantages, such as high sensitivity, selectivity, real-time spatial imaging, minimal damaging effects and simplicity of operation [9,10,11]. For these reasons, a number of fluorescent pH sensors have been developed based on the core fluorescent structures, such as 8-hydroxypyrene-1,3,6-trisulfonate (HPTS), fluorescein and rhodamine. However, the known fluorescent pH probes have some limitations. For example, the fluorescein-based probes are commonly known to be limited in photostability. And the HPTS derivatives are excited at relatively short wavelengths (<500 nm), thus they are interfered by high levels of auto-fluorescence and background scatterings. On the other hand, rhodamine-based probes have outstanding properties, such as high fluorescence quantum yields, good water solubility, and good photostability. In addition, it is well known that rhodamine derivatives are taken into cells without endocytosis because those dyes have positive charges, which show affinity for cell membranes with negative charges and endoplasmic reticula. Because of these properties, fluorescent rhodamine probes have been heavily utilized in biological imaging studies.

The equilibrium of rhodamine spirolactam between the colorless spirocyclic and fluorescent open forms is highly sensitive to pH environments. Thus, a lot of pH probes based on the rhodamine spirolactam have been developed [12,13,14,15,16,17,18,19,20,21,22,23,24,25,26]. The pH regulation of the spirolactam ring opening process could be modulated by introducing various factors, such as steric hindrance and hydrogen bonding groups [27,28,29,30,31,32,33,34,35,36,37,38,39]. Generally, amine derivatives of flexible aliphatic chains are introduced in the rhodamine spirolactams for the design of pH indicators, where the pKa values of the pH indicators are typically in the range of 3.0 to 5.2. And the positive charge of quaternary ammonium salt could be beneficial for the water solubility and pH responses of rhodamine amide probes [27].

**Scheme 1:**
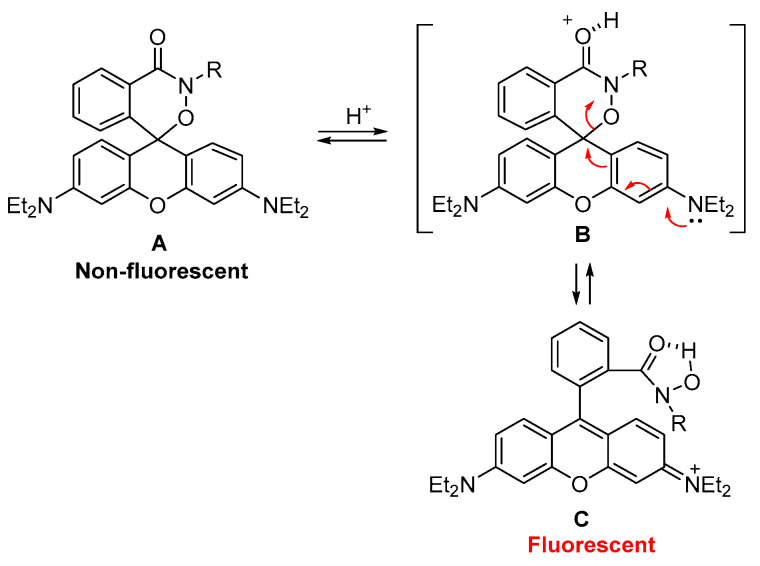
The proposed H^+^-sensing mechanism of rhodamine cyclic hydroxamate probe.

Since most of the known rhodamine pH probes are based on the five-membered spirolactam ring, we devised a rhodamine hydroxamate probe containing a six-membered spirolactam ring by using N–O instead of N for the lactam ring. Considering the pKa values of the hydroxamic acid [-CON(R)OH, pKa ~8.28] and the amide (-CONH, pKa ~15) [40], the six-membered cyclic hydroxamate spirolactam probe [41,42,43] with a N–O linkage is expected to have a lower pKa value compared to that of the five-membered lactam probes. To increase the water solubility of the pH probe, we introduced a tetraethylene glycol chain in the hydroxamate group. Thus, protonation on the carbonyl of the non-fluorescent rhodamine spirolactam form **A** would promote the ring opening to give the fluorescent opening form **C** as shown in Scheme 1. Thus, we envisioned that this probe could meet the pH range (pH 4.5–5.5) for the imaging of acidic lysosome-related organelles.

## 2. Results

### 2.1. Design and Synthesis of Probe ***1***

**Scheme 2:**
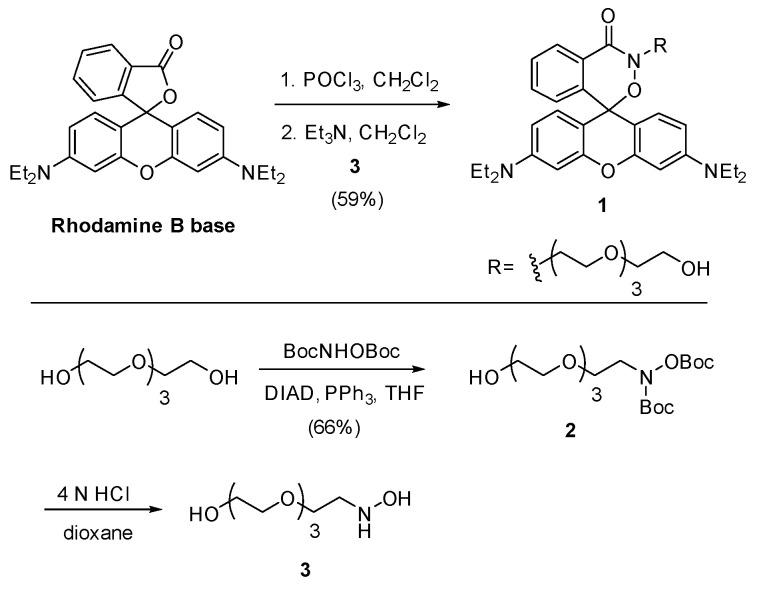
Synthesis of probe **1**.

The six-membered cyclic hydroxamate rhodamine probe **1** is designed to have a cyclic N–O linkage with a *N*-tetraethylene glycol chain to ensure water solubility (Scheme 2). Probe **1** was prepared from a rhodamine B base and tetraethylene glycol. Tetraethylene glycol was reacted with one equivalent of BocNHOBoc under Mitsubnobu conditions to give compound **2**. Then, the Boc groups were removed under strong acidic conditions using 4 N aqueous HCl solution to yield the tetraethylene glycol mono-hydroxylamine **3**. The fresh compound **3** was coupled with rhodamine B acid chloride, which is prepared from the rhodamine B base by using POCl_3_ in CH_2_Cl_2_, according to the known procedure, to give probe **1** [44]. Probe **1** was characterized using standard spectroscopic methods and shown to be in full agreement with the structure presented.

### 2.2. Optical Properties of Probe ***1***

**Figure 1:**
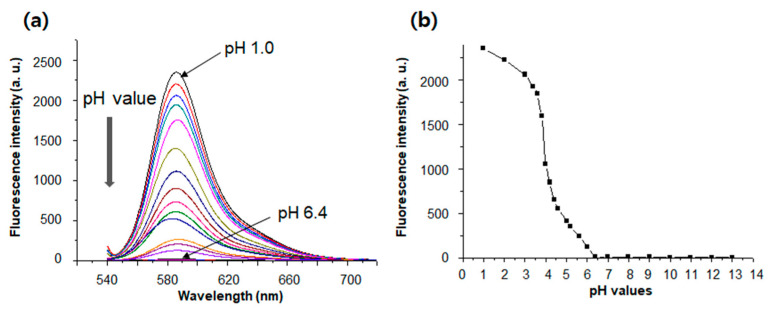
(**a**) Fluorescence intensity changes of probe **1** (2 μM) in aqueous solution (DMSO 1% *v*/*v*) of varying pH values (25 °C, Ex. 520 nm; Em. 585 nm). (**b**) Plot of fluorescence intensities at 585 nm depending on the different pH values. The aqueous solution (DMSO 1% *v*/*v*) of varying pH values were prepared using sodium chloride solution and sodium hydroxide solution. A solution of **1** (2.0 mL) was placed in a quartz cell (10.0 mm width) and the fluorescence spectrum was recorded. Fluorescence intensity changes (at 585 nm) were recorded after 30 min at 25 °C every time.

The synthesized probe **1** forms a colorless solution in H_2_O (DMSO 1% *v*/*v*) and does not show a strong fluorescent signal, indicating that the probe mainly exists in spirocyclic form. Thus, we measured the fluorescence intensity changes arising from the probe depending on various pH values (Figure 1). Aqueous solutions (DMSO 1% *v*/*v*, 25 °C) of probe **1** (2 μM) in different pH values were excited at 520 nm and the fluorescence intensities were recorded at 585 nm. Probe **1** shows strong fluorescence at low pH ranges (<pH 3) and fluorescence intensities decrease sharply around pH 3–5 range (Figure 1b). Then, it is non-fluorescent above pH 6, indicating it exists in the spirocyclic form predominantly as expected. The pKa of probe **1** from the fluorescence titration curve is calculated to be 4.1 via the analysis of fluorescence intensity changes as a function of pH using the Henderson–Hasselbalch equation (log [(I_max_ − I)/(I − I_min_)] = pH − pK_a_) (see Figure S1).

**Figure 2:**
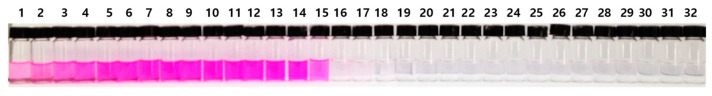
Color changes of probe **1** (20 μM) in aqueous solution (DMSO 1% *v*/*v*) of varying pH values: 1, pH = 1.0; 2, pH = 1.2; 3, pH = 1.4; 4, pH = 1.6; 5, pH = 1.8; 6, pH = 2.0; 7, pH = 2.2; 8, pH = 2.4; 9, pH = 2.6; 10, pH = 2.8; 11, pH = 3.0; 12, pH = 3.2; 13, pH = 3.4; 14, pH = 3.6; 15, pH = 3.8; 16, pH = 4.0; 17, pH = 4.2; 18, pH = 4.4; 19, pH = 4.6; 20, pH = 4.8; 21, pH = 5.0; 22, pH = 5.2; 23, pH = 5.4; 24, pH = 5.6; 25, pH = 5.8; 26, pH = 6.0; 27, pH = 7.0; 28, pH = 8.0; 29, pH = 9.0; 30, pH = 10.0; 31, pH = 11.0; and 32, pH = 12.0.

**Figure 3:**
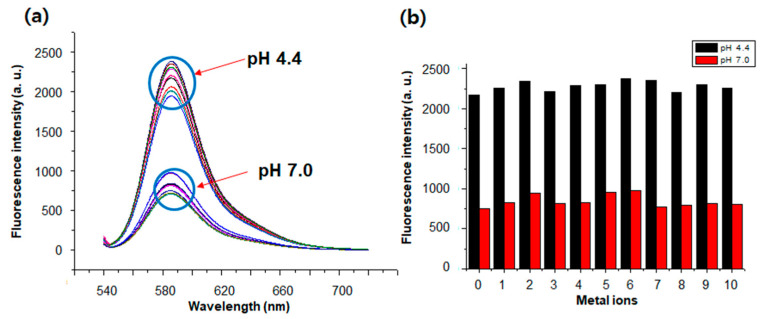
(**a**) Fluorescence spectra of **1** (2 μM) were recorded (aqueous DMSO 1% *v*/*v*) in the presence of metal ions (blank, Fe^3+^, Fe^2+^, Zn^2+^, Ca^2+^, Mn^2^, Mg^2+^, Cu^2+^, Na^+^, K^+^, Ba^2+^). (**b**) Plot of fluorescence intensities at 585 nm at pH = 4.4 and pH = 7.0 (aqueous DMSO 1% *v*/*v*) in the presence of metal ions (0, blank; 1, Fe^3+^; 2, Fe^2+^; 3, Zn^2+^; 4, Ca^2+^; 5, Mn^2+^; 6, Mg^2+^; 7, Cu^2+^; 8, Na^+^; 9, K^+^; 10, Ba^2+^). The aqueous solutions (DMSO 1% *v*/*v*) of different pH values were prepared using 0.1 M citric acid, 0.2 M sodium phosphate dibasic anhydrous, and 0.2 M sodium phosphate monobasic monohydrate. A solution of **1** (2.0 mL) was placed in a quartz cell (10.0 mm width) and the fluorescence spectrum was recorded. Fluorescence intensity changes (at 585 nm) were recorded after 30 min at 25 °C every time.

In addition to the fluorescence spectra, the solution of probe **1** undergoes distinct color changes from colorless to pink below pH ~ 4, indicating that probe **1** could serve as a “naked-eye” indicator (Figure 2 and Figure S2). To confirm the applicability of probe **1** in biological imaging studies, we examined the fluorescence intensities of probe **1** by the presence of biologically relevant metal ions at neutral and acidic pH conditions. As shown in Figure 3, the presence of biologically relevant metal ions (1, Fe^3+^; 2, Fe^2+^; 3, Zn^2+^; 4, Ca^2+^; 5, Mn^2+^; 6, Mg^2+^; 7, Cu^2+^; 8, Na^+^; 9, K^+^; 10, Ba^2+^) in the aqueous solution (DMSO 1% *v*/*v*) of probe **1** did not cause any significant fluorescent intensity changes at neutral pH (pH 7.0) and acidic pH (pH 4.4) conditions.

Probe **1** shows strong fluorescence intensities below pH 5.5 as shown in Figure 1b, and its fluorescence intensities at acidic pH conditions are not affected by the biologically relevant metal ions, as shown in Figure 3. And the calculated fluorescence quantum yield of probe **1** at pH 4.4 is 0.28 (Figures S3–S5). Therefore, we expected that probe **1** could be applicable for the imaging of acidic lysosome-related organelles with a pH range of 4.5–5.5.

### 2.3. Fluorescence and Imaging in Live PC3 and A549 Cells

**Figure 4:**
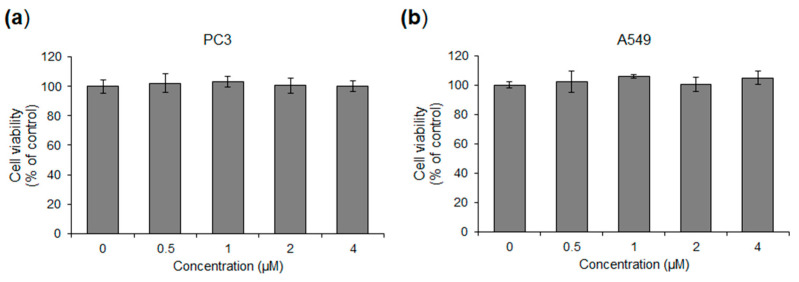
Cytotoxicity of probe **1** for living PC3 and A549 Cells. Cells were treated with the indicated concentration of probe **1**. Cell viability was measured using the EZ-Cytox colorimetric kit in PC3 (**a**) and A549 (**b**) cells (mean ± SD, n = 3).

To examine the biological applicability of probe **1** as an imaging probe for acidic organelles, we first determined the cytotoxicity of probe **1**. Cells were incubated with various concentrations of probe **1** for 2 h. The results indicated no cytotoxic effects in either PC3 or A549 cells (Figure 4). 

**Figure 5:**
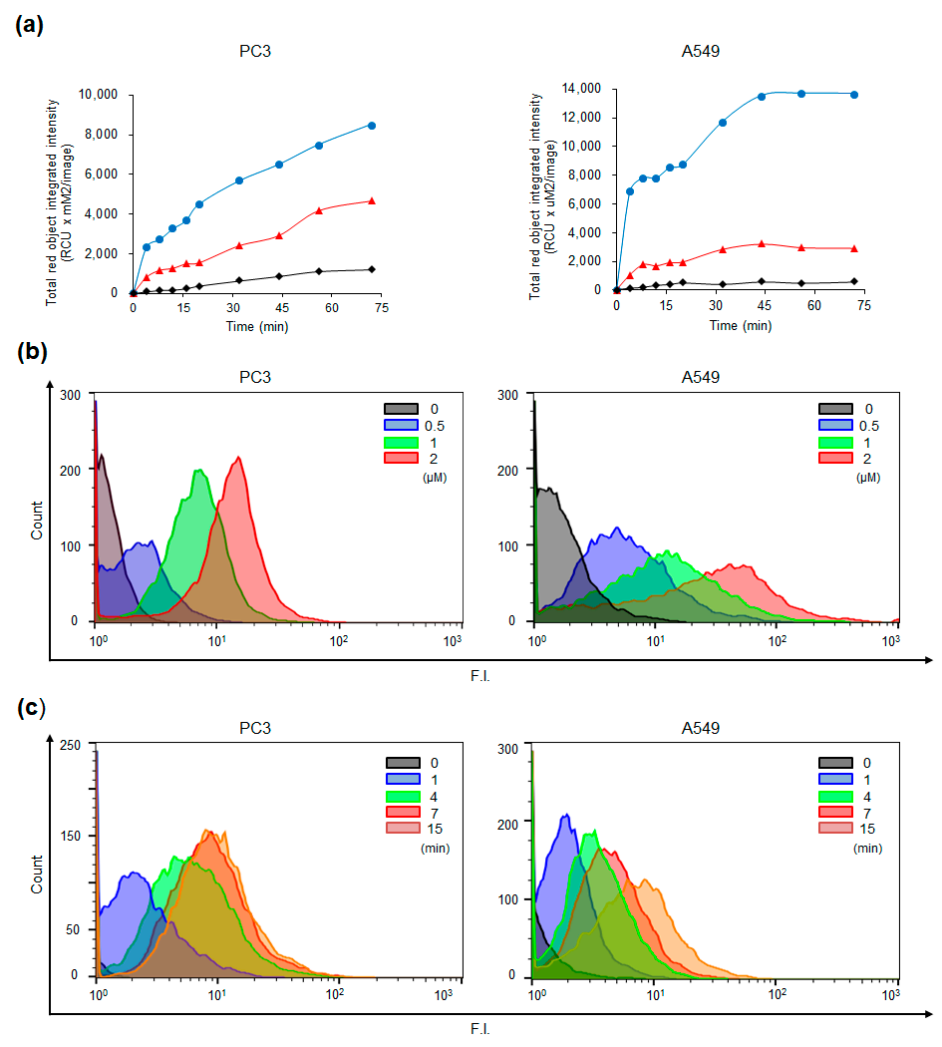
Real-time monitoring of changes in intracellular fluorescence. (**a**) PC-3 cells and (**b**) A549 cells were treated with various concentrations (0.5 (◆), 1 (▲), 2 (●) μM) of probe **1**. The fluorescence intensity was measured using the IncuCyte™ live content imaging system (Essen BioScience, Hertfordshire, UK). (**b**) PC3 and A549 cells were treated with various concentrations of probe **1** for a duration of 1 h. Subsequent to the 1 h incubation period, the intracellular fluorescence levels were measured using a flow cytometer. (**c**) Cells were treated with 1 μM of probe **1** for different time periods. After incubation for the indicated time, the intracellular fluorescence levels were measured using a flow cytometer.

Next, we evaluated the efficacy of the fluorescent pH probe **1** in lysosomes (pH 4.5–5.5) and lysosome-related organelles that provide an acidic environment [45,46]. First, we performed real-time monitoring of changes in intracellular fluorescence using the In-cuCyte™ live content imaging system (Essen BioScience, Hertfordshire, UK) (Figure 5a) [47]. PC3 and A549 cells were incubated with 0.5, 1 and 2 μM of probe **1** in a culture medium (DMEM) for 1 h 15 min at 37 °C. The fluorescence inside of the cells appeared in a short time and the fluorescence intensities increased concentration- and time-dependently. To assess the internalization fluorescence of probe **1**, we measured flow cytometry analysis. PC3 and A549 cells were treated with 0.5, 1 and 2 μM of probe **1** in culture medium (DMEM) for 1 h at 37 °C. The intracellular fluorescence of cells was observed at a low concentration, and its intensity increased in a concentration-dependent manner (Figure 5b). To further investigate the time period of internalization fluorescence of probe **1**, cells were treated with 1 μM of probe **1** for an indicated time period. Then, the fluorescence of cells was determined by using the flow cytometer. The fluorescence intensity inside the cells increased in a time-dependent manner. It was sufficient for the probe to internalize into the cells within 7 to 15 min (Figure 5c). These results demonstrate that probe **1** could be utilized effectively at low concentrations and that it rapidly internalizes into the cells.

**Figure 6:**
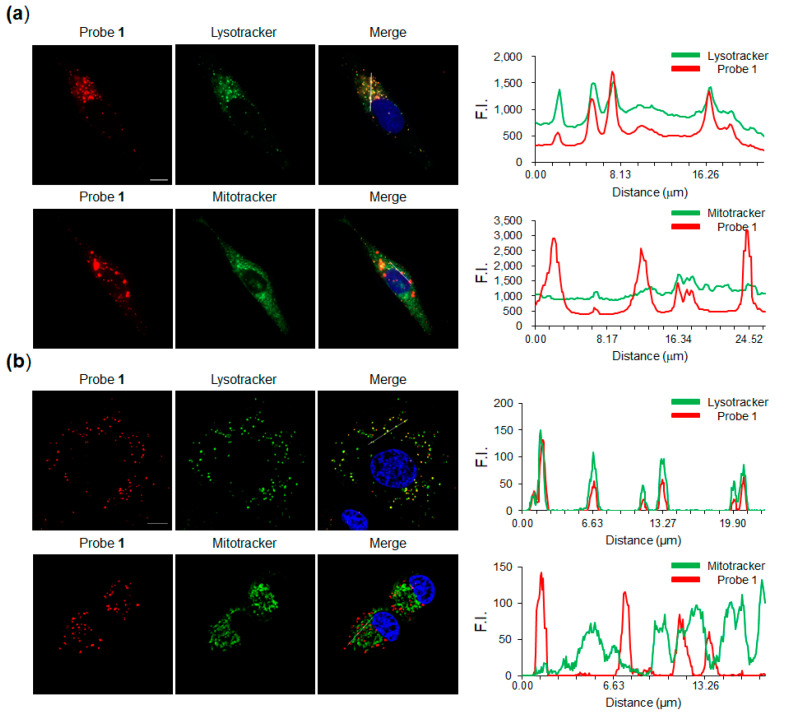
Fluorescence microscope images of living PC3 and A549 cells. (**a**) PC3 cells were co-strained with 1 μM of probe **1** and Lysotracker green or Mitotracker green for 1 h at 37 °C (Scale bar = 10 μm). (Pearson coefficient between probe **1** and lysotracker = 0.884; Pearson coefficient between probe **1** and mitotracker = 0.0647). (**b**) A549 Cells were co-strained with 1 μM of probe **1** and Lysotracker green or Mitotracker green for 1 h at 37 °C (Scale bar = 10 μm). (Pearson coefficient between probe **1** and lysotracker = 0.874; Pearson coefficient between probe 1 and mitotracker = −0.218). Graph shows correlation between probe 1 and FITC fluorescence in left images. Hoechst 33342, λ_ex_ = 405 nm, λ_em_ = 450 nm; Lysotracker green, λ_ex_ = 443 nm, λ_em_ = 505 nm; Mitotracker, λ_ex_ = 490 nm, λ_em_ = 516 nm; Probe **1**, λ_ex_ = 520 nm, λ_em_ = 585 nm.

To confirm the intracellular localization of the pH probe **1**, PC3 and A549 cells were co-stained with a fluorescent pH indicator, LysoSensorTM Green DND-189 (1 μM) and probe **1** (1 μM) in a culture medium for 1 h at 37 °C [48]. After staining the nucleus with Hoechst 33342 dye for 5 min, the remaining sensors were removed using PBS. As shown in Figure 6a, LysoSensorTM Green and pH sensors exhibited significant co-localization within lysosomes in PC3 cells. We could also observe the fluorescence, indicating lysosomes in another cell line, A549 (Figure 6b). Especially, the intracellular fluorescence patterns were observed at the lysosomes localized to the perinuclear region. Furthermore, co-staining with MytoTracker^®^ Green FM, which labels mitochondria, (10 nM) and 1 μM of probe **1** was performed to confirm the probe’s specific localization in the acidic organelles [49]. Unexpectedly, co-localized fluorescence patterns were not observed inside the cells. Moreover, the Pearson correlation coefficient between probe **1** and the Lysotracker showed a high correlation (Pearson’s correlation coefficient = 0.874). In contrast, the Pearson correlation coefficient between probe **1** and the MitoTracker showed a low or negative correlation (Pearson’s correlation coefficient = −0.218). These results strongly suggest that probe **1** could selectively stain acidic organelles such as lysosomes.

### 2.4. In Vivo Zebrafish Imaging

**Figure 7:**
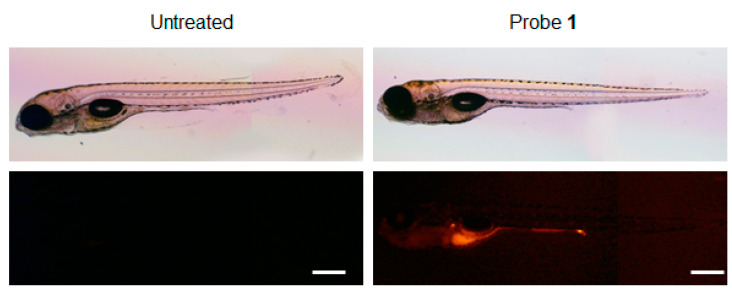
Microscopic and fluorescent images of zebrafish. Images of 5 dpf zebrafish were treated with 1 μM probe **1** for 1 h. The images were obtained using fluorescence microscope. (**Top**, phase contrast image; **bottom**, fluorescence image). Red fluorescence: λ_ex_ = 520 nm, λ_em_ = 585 nm. Scale bars = 500 μm.

To further confirm the applicability of probe **1**, we then tested zebrafish as a whole-organism model to evaluate if pH probe **1** could be utilized to detect acidic organs in living organisms. Five-day-old zebrafish were exposed to 1 μM probe **1,** which was diluted in E3 embryo media for a duration of 1 h. After a thorough rinsing with fresh media, the zebrafish were imaged using fluorescence microscopy. As expected, the strong fluorescence signal of probe **1** appeared in the intestine selectively (Figure 7).

## 3. Discussion

In conclusion, we have successfully developed a novel rhodamine-based cyclic hydroxamate derivative as a fluorescent pH probe, which is applicable in aqueous media, in live cells, such as PC3, A549 cells, and in zebrafish. We were able to tune the pH range where the ring opening and closing of the spirolactam occurs using a six-membered N–O spirolactam ring of rhodamine B. The novel cyclic hydroxamate structure of rhodamine B shows a pKa ~4.1 value and strong fluorescence intensities below pH ~ 5.5. Therefore, it is ideal for monitoring acidic lysosome-related organelles with a pH range of 4.5–5.5.

Thus, we demonstrated the practical utility of the pH probe **1** by monitoring pH changes effectively within lysosomes in live PC3, A549 cells and zebrafish. The probe exhibited a high degree of selectivity for lysosomal labeling. Therefore, we believe that this pH probe could find significant applications in cell imaging and the monitoring of pH fluctuations within lysosomes and other related acidic organelles.

In summary, the novel probe **1** is highly fluorescent in acidic conditions and it could have significant potential applications in various fields, such as live cell imaging of an acidic environment and the precise tracking of pH dynamics within lysosomes and related acidic organelles.

## 4. Materials and Methods

### 4.1. Synthesis of Probe ***1***

Synthesis of **2**: To a solution of tetraethylene glycol (0.888 mL, 5.15 mmol) in THF (5.0 mL), we added triphenylphosphine (743 mg, 2.83 mmol) and *N*,*O*-Di-Boc-hydroxylamine (600 mg, 2.57 mmol) in THF (5 mL) at 0 °C. To this mixture, we added dropwise diisopropyl azodicarboxylate (0.56 mL, 2.83 mmol) at room temperature. The reaction was stirred for 12 h. The solvent was evaporated under vacuum and the crude product was purified using flash column chromatography (CH_2_Cl_2_/MeOH = 150:1 to 30:1) to give 1.4 g (66%) of **2** as pale yellow liquid: ***R*_f_** = 0.34 (DCM/MeOH = 20:1); **^1^H NMR** (400 MHz, CDCl_3_) δ = 3.75–3.54 (m, 16 H), 2.71 (s, 1 H), 1.48 (s, 9 H), 1.43 (s, 9 H); **^13^C NMR** (400 MHz, CDCl_3_) δ = 154.7, 152.2, 84.7, 82.3, 72.5, 70.6, 70.4, 70.4, 70.3, 67.6, 61.6, 49.6, 28.0, 27.6; **IR** (film, cm^−1^): 3452, 2979, 2934, 2872, 1783, 1716, 1477, 1459, 1394, 1369, 1255, 1232, 1125, 1069; **HRMS** *m/z* calcd. for C_18_H_35_N_1_O_9_ [(M + H)^+^]: 410.2300; found: 410.2374.

Synthesis of probe **1:** To a solution of rhodamine B base (297 mg, 0.671 mmol) in CH_2_Cl_2_ (3.4 mL), we added phosphorus oxychloride (0.31 mL, 3.36 mmol) dropwise over 2 min. The mixture was heated under reflux for 3 h. The reaction mixture was cooled to room temperature. The volatile materials were removed under reduced pressure to give rhodamine B acid chloride, which was used for the next step without purification.

Compound **2** (330 mg, 0.806 mmol) was dissolved in 4M HCl in 1,4-dioxane (4.0 mL). The reaction mixture was stirred for 24 h. After the reaction, solvent was evaporated under vacuum to give **3,** which was used for next step reaction without further purification.

To a freshly prepared rhodamine B acid chloride (335 mg, 0.671 mmol) in CH_2_Cl_2_ (3.4 mL), we added **3** (168 mg, 0.806 mmol) in CH_2_Cl_2_ (2.0 mL) solution. After 5 min, to the reaction mixture, we added slowly Et_3_N (0.748 mL, 5.368 mmol) at 0~5 °C. The reaction mixture was stirred for 2 h at room temperature. The solution was diluted with water, extracted with CH_2_Cl_2_ several times and washed with saturated aqueous ammonium chloride. The combined organic layers were dried over anhydrous MgSO_4_, filtered, and concentrated under reduced pressure. And the crude product was purified using flash column chromatography (CH_2_Cl_2_:MeOH = 150:1 to 40:1) to give 251 mg (59%) of probe **1** as a red oil: ***R*_f_** = 0.35 (CH_2_Cl_2_/MeOH = 20:1); **^1^H NMR** (400 MHz, CDCl_3_) δ = 8.21 (m, 1 H), 7.45 (m, 2 H), 7.01 (m, 1 H), 6.84 (m, 2 H), 6.47 (s, 2 H), 6.33 (m, 2 H), 3.70–3.60 (m, 4 H), 3.60–3.50 (m, 6 H), 3.50(m, 2 H), 3.40–3.30 (m, 10 H), 3.14 (t, *J* = 6.4 Hz, 2 H), 2.62 (s, 1 H), 1.17 (t, *J* = 6.8 Hz, 12 H); **^13^C NMR** (400 MHz, CDCl_3_) δ = 163.4, 153.7, 149.5, 141.7, 132.4, 131.0, 128.5, 127.9, 127.4, 127.0, 107.0, 107.0, 97.8, 80.5, 72.6, 70.7, 70.5, 70.4, 70.3, 66.6, 61.9, 46.3, 44.6, 12.7; **IR** (film, cm^−1^): 3437, 2926, 1736, 1613, 1517, 1429, 1356, 1222, 1123; **HRMS (FAB)** *m/z* calcd. for C_36_H_47_N_3_O_7_ [(M + H)^+^]: 634.3400; found: 634.3470.

### 4.2. Cytotoxicity of Probe ***1***

PC-3 (human prostate adenocarcinoma) and A549 (human lung adenocarcinoma epithelial cells) obtained from American Type Culture Collection (Manassas, VA, USA) were maintained in Dulbecco’s Modified Eagle’s Medium (DMEM) supplemented with 10% fetal bovine serum (FBS), 50 units/mL of penicillin and 50 μg/mL of streptomycin. To measure cytotoxicity of probe **1**, PC-3 and A549 cells were seeded in a 96-well plate at a density of 1.5 × 10^4^ cells per well in culture media. After 16 h, the cells were treated with various concentrations of probe **1** in culture media containing 0.1% (*v*/*v*) dimethyl sulfoxide (DMSO) for 2 h. A total of 10 μL of Ez-Cytox solution was directly added into each well and further incubated for 2 h. The absorbance of samples was obtained using a microplate reader (Infinite 200 pro, TECAN, Männedorf, Switzerland).

### 4.3. Real-Time Monitoring of Changes in Intracellular Fluorescence

PC3 (human prostate adenocarcinoma) and A549 (human lung adenocarcinoma epithelial cells) obtained from American Type Culture Collection (Manassas, VA, USA) were maintained in DMEM (Dulbecco’s Modified Eagle’s Medium) supplemented with 10% fetal bovine serum (FBS), 50 units/mL of penicillin and 50 μg/mL of streptomycin. PC3 and A549 cells were seeded in a 24-well plate at a density of 3 × 10^4^ cells per well in culture media. After 16 h, the cells were treated with various concentrations of probe **1** in culture media containing 0.1% (*v*/*v*) DMSO and fluorescence intensity was determined using a IncuCyte™ live content imaging system (Essen BioScience, Hertfordshire, UK).

### 4.4. Measurement of Intracellular Fluorescence in a Dose-Dependent or Time-Dependent Manner

PC3 and A549 cells were seeded in a 6-well plate at a density of 1.5 × 10^5^ cells per well in culture media. After 16 h, the cells were treated with various concentrations of probe **1** in culture media containing 0.1% (*v*/*v*) DMSO for a 1 h. After incubation, cells were washed with Dulbecco’s phosphate-buffered saline (DPBS) and harvested using a trypsin-EDTA solution. For time-dependent analysis, cells were incubated with 1 μM of probe 1 for various time points. Cells were re-suspended in DPBS and observed using a flow cytometer (BD Biosciences, East Rutherford, NJ, USA). The results were analyzed using FlowJo v11 software (BD Biosciences).

### 4.5. Imaging of Mammalian Cells Incubated with pH Probe

PC3 and A549 cells were seeded in a 24-well plate at a density of 3 × 10^4^ cells per well in DMEM media supplemented with 10% FBS, 100 units/mL of penicillin and 100 μg/mL. After 16 h, PC3 and A549 cells were incubated with various concentrations of probe **1** in culture media for 1 h, respectively. LysoSensorTM Green DND-189 (1 μM) (Cat. L7535, Invitrogen, Waltham, MA, USA) was used as the positive control that appears in acidic conditions. Also, cells were co-stained with Mitochondrion-selective probe, MitoTracker^®^ Green (10 nM) (Cat. M7514, Invitrogen). Nucleus staining with Hoechst 33342 was used to ensure for viability of cells. After washing with PBS, the treated cells were analyzed using confocal microscopy (LSM 710, Carl Zeiss, Germany). (Hoechst 33342, λ_ex_ = 405 nm, λ_em_ = 450 nm; Lysotracker green, λ_ex_ = 443 nm, λ_em_ = 505 nm; Mitotracker, λ_ex_ = 490 nm, λ_em_ = 516 nm; Probe **1**, λ_ex_ = 520 nm, λ_em_ = 585 nm).

### 4.6. Imaging of Zebrafish Incubated with pH Probe

Zebrafish were kept in a circulating system at 28.5 °C and fed with brine shrimp twice a day. For mating, male and female zebrafish were maintained in one tank at 28.5 °C on a 14 h light/10 h dark cycle and then the spawning of eggs was triggered by giving light stimulation in the morning. Almost all the eggs were fertilized immediately. The zebrafish larvae at 5 dpf (days post fertilization) were maintained in E3 embryo media (5 mM NaCl, 0.17 mM KCl, 0.33 mM MgSO_4_, 0.33 mM CaCl_2_, 10–5% methylene blue; pH 7.5). In the staining experiment, the larvae at 5 dpf were exposed to 1 μM of probe **1** diluted in E3 media for 1 h. After washing with fresh media, the zebrafish were imaged using fluorescence microscopy (TE2000, Nikon, Japan).

### 4.7. Statistical Analysis

All data are expressed as mean ± standard deviation (SD) of three or more independent experiments. The statistical analyses were conducted using GraphPad Prism software (GraphPad 8.4.3, San Diego, CA, USA). 

## Data Availability

Not applicable.

## Supplementary Materials

The following supporting information can be downloaded at: https://www.mdpi.com/article/10.3390/ijms242015073/s1. References [50,51,52,53] are cited in Supplementary Materials.
