# Supplementary material for: Rhodamine-Based Cyclic Hydroxamate as Fluorescent pH Probe for Imaging of Lysosomes
Source: Int J Mol Sci. 2023 Oct 11;24(20):15073. doi: 10.3390/ijms242015073 (PMC10606023; doi:10.3390/ijms242015073)

Supplementary Materials

Rhodamine-based cyclic hydroxamate as fluorescent pH probe for imaging of lysosomes

Young Ju Kim[1,†], Mina Jang[2,3,†], Jongtae Roh[2,4,†], Yoon Jeong Lee[1], Hee Jung Moon[1], Jimin Byun[1], Jihyun Wi[1], Sung-Kyun Ko[2,4]* and Jinsung Tae[1,]*

[1] Department of Chemistry, Yonsei University, Seoul 03722, Korea;

[2] Chemical Biology Research Center, Korea Research Institute of Bioscience and Biotechnology (KRIBB), Cheongju 28116, Korea

[3] New Drug Development Center, Daegu-Gyeongbuk Medical Innovation Foundation, Daegu 41061, Korea

[4] KRIBB School of Bioscience, Korea University of Science and Technology (UST), Daejeon 34141, Korea

CONTENTS

1. General methods………………………………………………………… S2

2. Synthesis ……………………………………………………………… S3

3. Cell Study …………………………………………………………….. S10

4. NMR spectra …………………………………………………………….. S12

1. General methods

General synthetic materials and methods: Silica gel 60 (230-400 mesh, Merck) was used for column chromatography. Analytical thin layer chromatography was performed using Merck 60 F254 silica gel (pre-coated sheets, 0.25 mm thick). All reagents and solvents were purchased from Sigma-Aldrich, TCI, Alfa and Acros, and used as received with the following exceptions. Dichloromethane (CH_2Cl_2) was distilled from calcium hydride (CaH_2) and tetrahydrofuran (THF) from sodium (Na) and benzophenone.

Spectroscopic materials and methods: Nuclear magnetic resonance (NMR) spectra were recorded in $CDCl_3$ unless stated otherwise with internal references ($CHCl_3$, δ = 7.26 ppm and TMS, δ = 0.00 ppm) at ambient temperature mainly on Bruker II-400 Fourier Transform Spectrometers operating at 400 MHz for ^1H and at 100 MHz for ^{13}C. Mass spectra were recorded on an Ultimate 3000 RS-Q-Exactive Orbitrap Plus mass spectrometer for both low resolution and high resolution mass spectra. The pH was recorded by HI-8014 instrument (HANNA). Infrared absorption spectra were recorded as a KBr pellet on a Vertex 70 FT-IR spectrophotometer. All UV−Vis spectra were measured with UV−visible spectrophotometer (V-650). Fluorescence spectrophotometer (LS 55) was used to obtain fluorescence emission spectrum in liquid state. The slit width was 10 nm for both excitation and emission. Samples were included in a 10.0 mm long quartz cuvette (3.5 ml volume). The photon multiplier voltage was 400 V and a circulating PBS buffer/DMSO bath was used during all experiments to regulate the temperature at 37 ^oC. The excitation-time emission spectrum at 520 nm was integrated over the range of 540-720 nm. All measurements were performed at least three times.

2. Synthesis

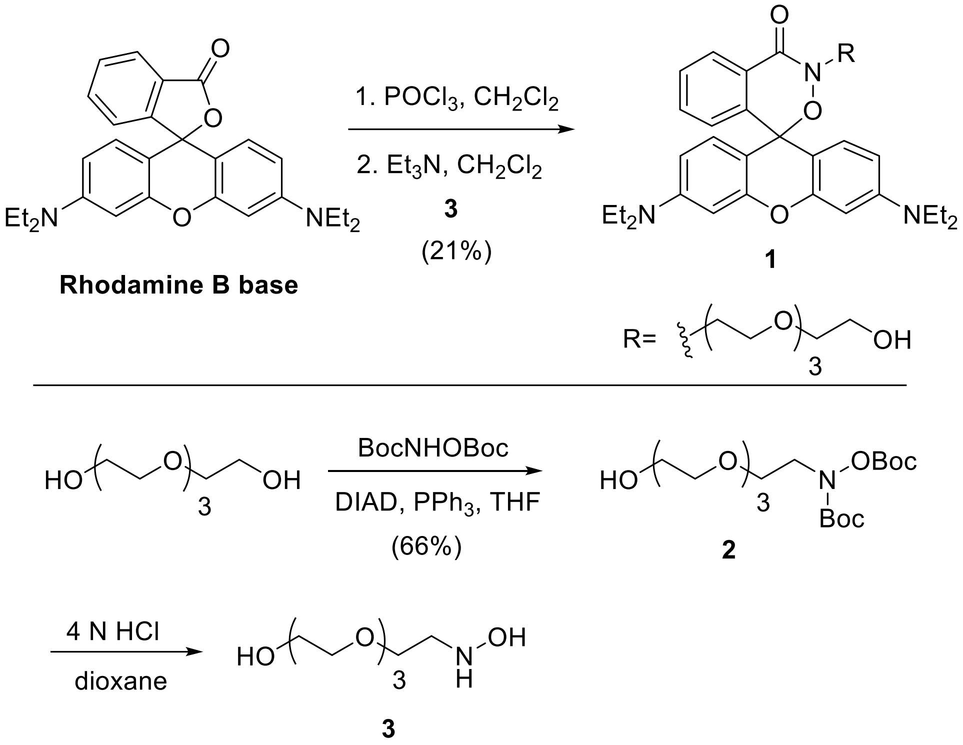

Compound 2: To a solution of tetraethylene glycol (0.888 mL, 5.15 mmol) in THF (5.0 mL) was added triphenylphosphine (743 mg, 2.83 mmol) and *N*,*O*-Di-Boc-hydroxylamine (600 mg, 2.57 mmol) in THF (5 ml) at 0 °C. To this mixture was added dropwise diisopropyl azodicarboxylate (0.56 mL, 2.83 mmol) at room temperature. The reaction was stirred for 12 hr. The solvent was evaporated under vacuum and the crude product was purified by flash column chromatography (CH_2Cl_2/ MeOH = 150 : 1 to 30 : 1) to give 1.4 g (66 %) of 2 as pale yellow liquid: $\boldsymbol{R}_f$ = 0.34 (DCM / MeOH = 20 : 1); ^{1}**H NMR** (400 MHz, $CDCl_3$) δ = 3.75-3.54 (m, 16 H), 2.71 (s, 1 H), 1.48 (s, 9 H), 1.43 (s, 9 H); ^{13}**C NMR** (400 MHz, $CDCl_3$) δ = 154.7, 152.2, 84.7, 82.3, 72.5, 70.6, 70.4, 70.4, 70.3, 67.6, 61.6, 49.6, 28.0, 27.6; **IR** (film, cm^{-1}): 3452, 2979, 2934, 2872, 1783, 1716, 1477, 1459, 1394, 1369, 1255, 1232, 1125, 1069; **HRMS** *m/z* calcd. for $C_{18}H_{35}N_1O_9$ [$(M + H)^+$]: 410.2300; found: 410.2374.

Probe 1: To a solution of rhodamine B base (297 mg, 0.671 mmol) in CH_2Cl_2 (3.4 mL) was added phosphorus oxychloride (0.31 mL, 3.36 mmol) dropwise over 2 min. The mixture was heated under reflux for 3 h. The reaction mixture was cooled to room temperature. The volatile materials were removed under reduced pressure to give rhodamine B acid chloride, which was used for the next step without purification.

Compound **2** (330 mg, 0.806 mmol) was dissolved in 4M HCl in 1,4-dioxane (4.0 mL). The reaction mixture was stirred for 24 hr. After the reaction, solvent was evaporated under vacuum to give **3** which was used for next step reaction without further purification.

To a freshly prepared rhodamine B acid chloride (335 mg, 0.671 mmol) in CH_2Cl_2 (3.4 mL) was added **3** (168 mg, 0.806 mmol) in CH_2Cl_2 (2.0 mL) solution. After 5 minutes, to the reaction mixture was added slowly Et_3N (0.748 mL, 5.368 mmol) at 0~5℃. The reaction mixture was stirred for 2 hr at room temperature. The solution was diluted with water, extracted with CH_2Cl_2 several times and washed with saturated aqueous ammonium chloride. The combined organic layers were dried over anhydrous $MgSO_4$, filtered, and concentrated under reduced pressure. And the crude product was purified by flash column chromatography (CH_2Cl_2 : MeOH = 150 : 1 to 40 : 1) to give 251 mg (59 %) of probe **1** as an red oil: $\boldsymbol{R_f}$ = 0.35 (CH_2Cl_2/ MeOH = 20 : 1); **^{1}H NMR** (400 MHz, $CDCl_3$) δ = 8.21 (m, 1 H), 7.45 (m, 2 H), 7.01 (m, 1 H), 6.84(m, 2 H), 6.47 (s, 2 H), 6.33 (m, 2 H), 3.70-3.60 (m, 4 H), 3.60-3.50 (m, 6 H), 3.50(m, 2 H), 3.40-3.30 (m, 10 H), 3.14 (t, *J*=6.4 Hz, 2 H), 2.62 (s, 1 H), 1.17 (t, *J*= 6.8 Hz, 12 H); **^{13}C NMR** (400 MHz, $CDCl_3$) δ = 163.4, 153.7, 149.5, 141.7, 132.4, 131.0, 128.5, 127.9, 127.4, 127.0, 107.0, 107.0, 97.8, 80.5, 72.6, 70.7, 70.5, 70.4, 70.3, 66.6, 61.9, 46.3, 44.6, 12.7; **IR** (film, cm^{-1}): 3437, 2926, 1736, 1613, 1517, 1429, 1356, 1222, 1123; **HRMS (FAB)** *m/z* calcd. for $C_{36}H_{47}N_3O_7$ $[(M + H)^+]$: 634.3400 ; found: 634.3470.

Structure Confirmation of Probe 1

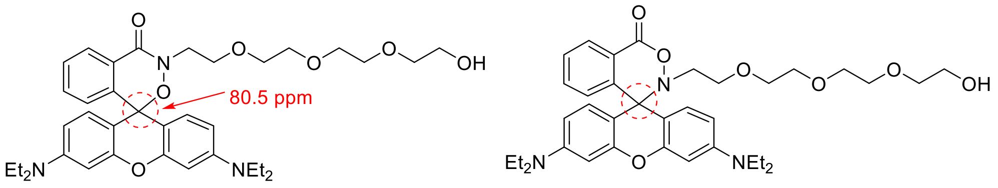

1 E1

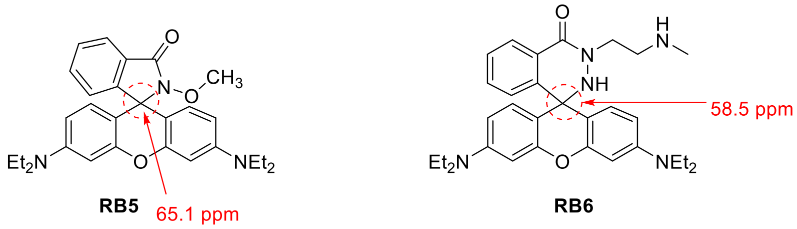

RB5 RB6

Tetrahedron Lett. **2007**, *48(31)*, 5389-5392

RSC Adv., **2022**, *12*, 22435–22439

The structure of probe **1** is confirmed by comparison the chemical shift of spiro-carbon with the reported rhodamine spirolactam derivatives in the literature. The reported spiro-carbon chemical shifts of 5- and 6-membered rhodamine spirolactams (**RB5** and **RB6**) [50, 41] show that the chemical shifts of "***N***"-bonded spiro-carbons are 58.5 ppm (**RB6**) and 65.1 ppm (**RB5**), which are consistent with other rhodamine spirolactams derivatives in the literatures. Meanwhile, the spiro-carbon chemical shift of the isolated product **1** is 80.5 ppm, which is highly downfield shifted due to the electronegative oxygen atom. This could imply "***O***"-bonded spiro-carbon (probe **1**) instead "***N***"-bonded spiro-carbon (**E1**).

Calculation of pKa of probe 1 by fluorescence titration curve.

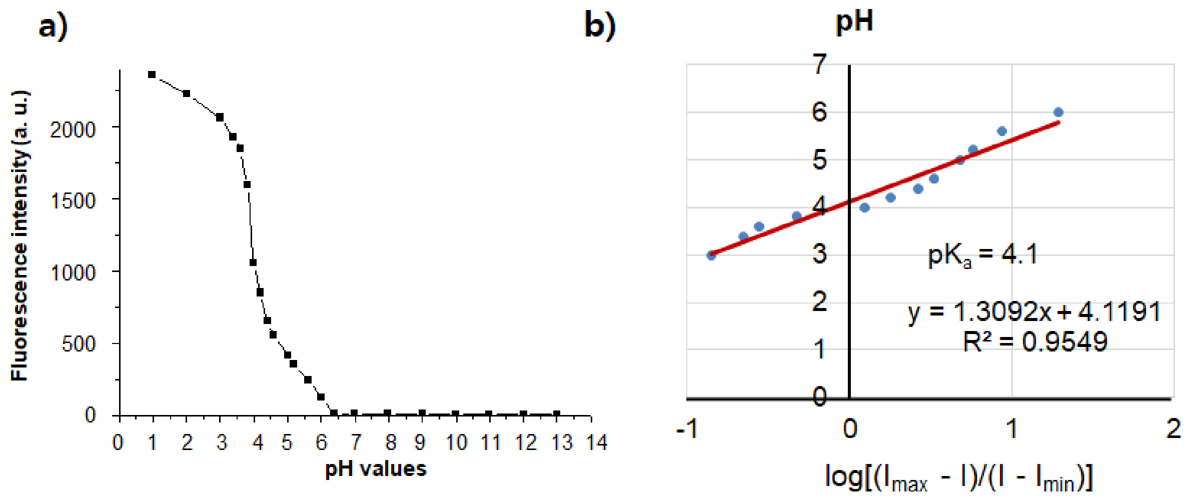

Figure S1. (a) Plot of fluorescence intensities at 585 nm depending on the different pH values. Fluorescence intensity changes of probe **1** (2 μM) in aqueous solution (DMSO 1% v/v) of varying pH values (25 °C, Ex. 520 nm; Em. 585 nm). The aqueous solution (DMSO 1% v/v) of varying pH values were prepared using sodium chloride solution and sodium hydroxide solution. A solution of **1** (2.0 mL) was placed in a quartz cell (10.0 mm width) and the fluorescence spectrum was recorded. Fluorescence intensity changes (at 585 nm) were recorded after 30 min at 25 °C every time. (b) The fitting plot for fluorescence titration against pH. (b)

The pKa of probe **1** was calculated by the analysis of fluorescence intensity changes as a function of pH by using the Henderson-Hasselbalch equation: $\log[(I_{max}-I)/(I-I_{min})] = \mathrm{pH}-\mathrm{p}K_a$ where I is the observed fluorescence intensity, I_{max} and I_{min} are the corresponding maximum and minimum respectively [51]. The calculated $\mathrm{p}K_a$ of sensor **1** is 4.1.

UV-Vis absorption of probe 1 (7 µM) in DMSO 1% v/v.

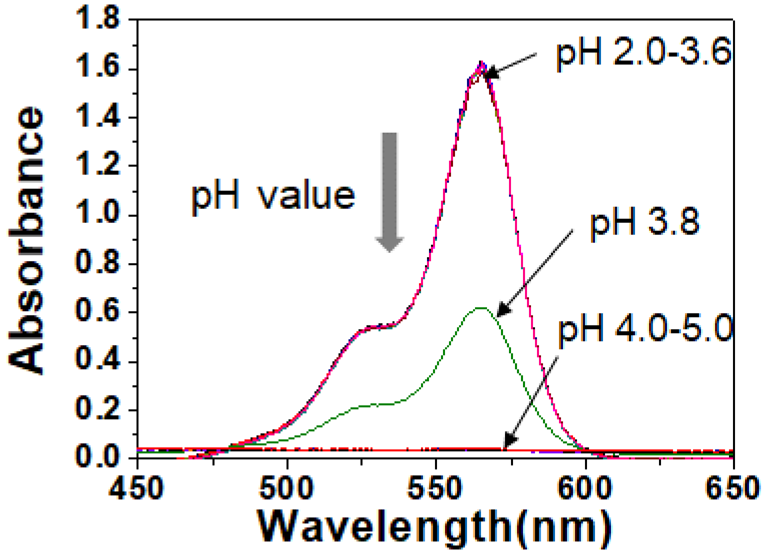

Figure S2. UV-Vis absorption of probe **1** (20 µM) in DMSO 1% v/v. Absorption titration of **1** (20 µM) in varying pH values (pH values = 2.0, 2.2, 2.4, 2.6, 2.8, 3.0, 3.2, 3.4, 3.6, 3.8, 4.0, 4.2, 4.4, 4.6, 4.8, 5.0) were recorded in H_2O (DMSO 1% v/v). The water solution (DMSO 1% v/v) of varying pH values were prepared using hydrochloric acid solution and sodium hydroxide solution. A solution of **1** (2.0 mL) was placed in a quartz cell (10.0 mm width) and the absorption spectrum was recorded. Each UV-curves was recorded after 30 min at 25 °C every time.

Fluorescence quantum yield of probe 1 (2 µM) at pH 4.4 in DMSO 1% v/v.

A pH 4.4 aqueous solution of probe **1** (4µM) in DMSO (1% v/v) was prepared by 0.1M citric acid. Rhodamine B in water (Φ_{RB}= 0.31 at λ_{ex}= 546 nm) was used as a standard fluorophore [52].

The absorption and emission spectra of probe **1** and rhodamine B were obtained at different concentration (Figure S3 and S4).

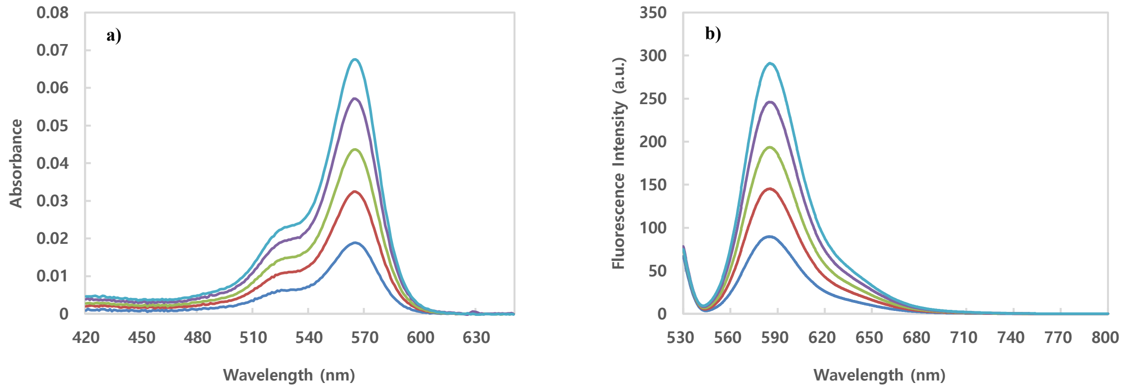

Figure S3. Absorption (a) and emission (b) spectra of probe **1** in aqueous 1% DMSO obtained at different concentrations (excitation wavelength at 520 nm).

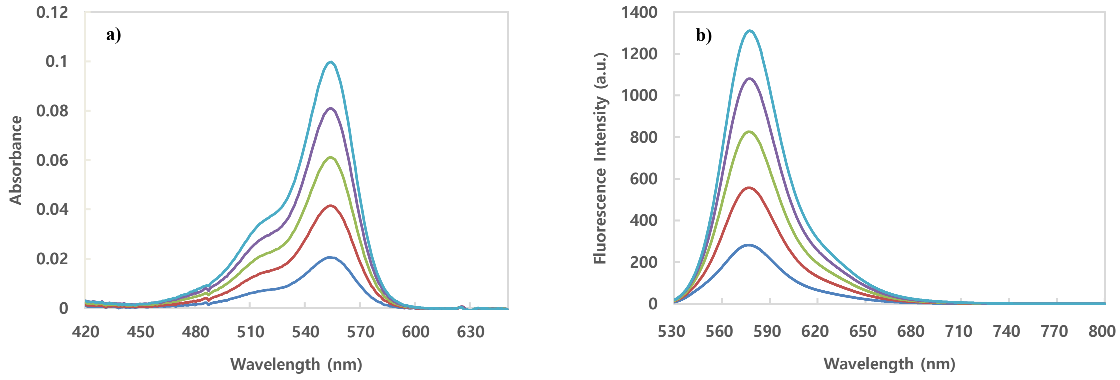

Figure S4. Absorption (a) and emission (b) spectra of rhodamine B in water obtained at different concentrations (excitation wavelength at 546 nm).

The following equation has been used for calculation of the values of fluorescence quantum yield [53];

$$\Phi_X = \Phi_{RB}\left(\frac{Grad_X}{Grad_{RB}}\right)\left(\frac{n_X^2}{n_{RB}^2}\right) \qquad (1)$$

Where Φ_{RB} is fluorescennce quantum yield of rhodamine B, n_x, n_{RB} are the refractive indexes of used solvents (aqueous 1% DMSO and water, respectively) and $Grad_X$ and $Grad_{RB}$ are the gradients from the plots of integrated fluorescence intensity versus absorbance (Figure S5).

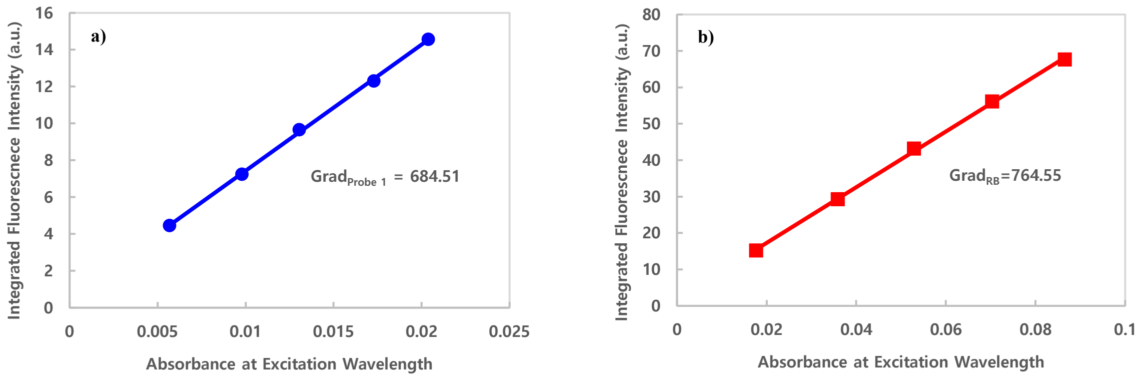

Figure S5. The dependences of integrated fluorescence intensity on absorbance values for (a) probe **1** and (b) rhodamine B with extracted values of gradients.

According to Eq. 1, the calculated values of fluorescence quantum yield of probe **1** at pH 4.4 (excitation wavelength 520 nm) was found to be $\Phi_{probe\ 1} = 0.28$.

References

41. Kim, S. Y.; Park, S.-H.; Lee, C.-H.; Tae, J.; Shin, I. *RSC Adv*. **2022**, *12*, 22435-22439.
50. Bae, S.; Tae, J. *Tetrahedron Lett.* **2007**, *48*, 5389-5392.
51. Henderson, L.J. *Am. J. Physiol*. **1908**, *21*, 173–179.
52. Magde, D.; Rojas ,G.E.; Seybold, P. *Photochem. Photobiol*. **1999**, *70*, 737-744.
53. Williams, A.T.R.; Winfield, S.A.; Miller, J. N. *Analyst* **1983**, *108*, 1067-1071.

3. Cell Study

3.1. Real-time monitoring of changes in intracellular fluorescence.

PC-3 (human prostate adenocarcinoma) and A549 (human lung adenocarcinoma epithelial cells) obtained from American Type Culture Collection (Manassas, VA) were maintained in DMEM (Dulbecco's Modified Eagle Medium) supplemented with 10% fetal bovine serum (FBS), 50 units/mL of penicillin and 50 μg/mL of streptomycin. PC-3 and A549 cells were seeded in a 24-well plate at a density of 3 x 10^4 cells per well in culture media. After 16 h, the cells cells were treated with various concentrations of probe **1** in culture media containing 0.1% (v/v) DMSO and fluorescence intensity was determined using a IncuCyte™ live content imaging system (Essen BioScience, Hertfordshire, UK).

3.2. Imaging of mammalian cells incubated with pH probe.

PC-3 and A549 cells were seeded in a 24-well plate at a density of 3 x 10^4 cells per well in DMEM media supplemented with 10% FBS, 100 units/mL of penicillin and 100 μg/mL). After 16 h, PC-3 and A549 cells were incubated with various concentrations of probe **1** in culture media for 1 h, respectively. LysoSensorTM Green DND-189 (1 μM) (Cat. L7535, Invitrogen) was used as the positive control that appears in acidic conditions. Also, cells were co-stained with Mitochondrion-selective probe, MitoTracker® Green (10 nM) (Cat. M7514, Invitrogen). Nucleus staining with Hoechst 33342 was used to ensure for viability of cells. After washing with PBS, the treated cells were analyzed by using confocal microscopy (LSM 710, Carl Zeiss, Germany).

3.3. Imaging of zebrafish incubated with pH probe.

Zebrafish were kept in a circulating system at 28.5 ºC and fed with brine shrimps twice a day. For mating, male and female zebrafish were maintained in one tank at 28.5 ºC on a 14 h light/10 h dark cycle and then the spawning of eggs were triggered by giving light stimulation in the morning. Almost all the eggs were fertilized immediately. The zebrafish larvae at 5 dpf (days post fertilization) were maintained in E3 embryo media (5 mM NaCl, 0.17 mM KCl, 0.33 mM $MgSO_4$, 0.33 mM $CaCl_2$, 10-5% methylene blue; pH 7.5). In the staining experiment, the larvae

at 5 dpf were exposed to 1 μM of probe **1** diluted in E3 media for 1 h. After washing with fresh media, the zebrafish were imaged by fluorescence microscopy (TE2000, Nikon, Japan).

4. NMR spectra

4.1. Compound 2

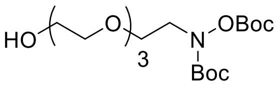

2

^{1}H-NMR data

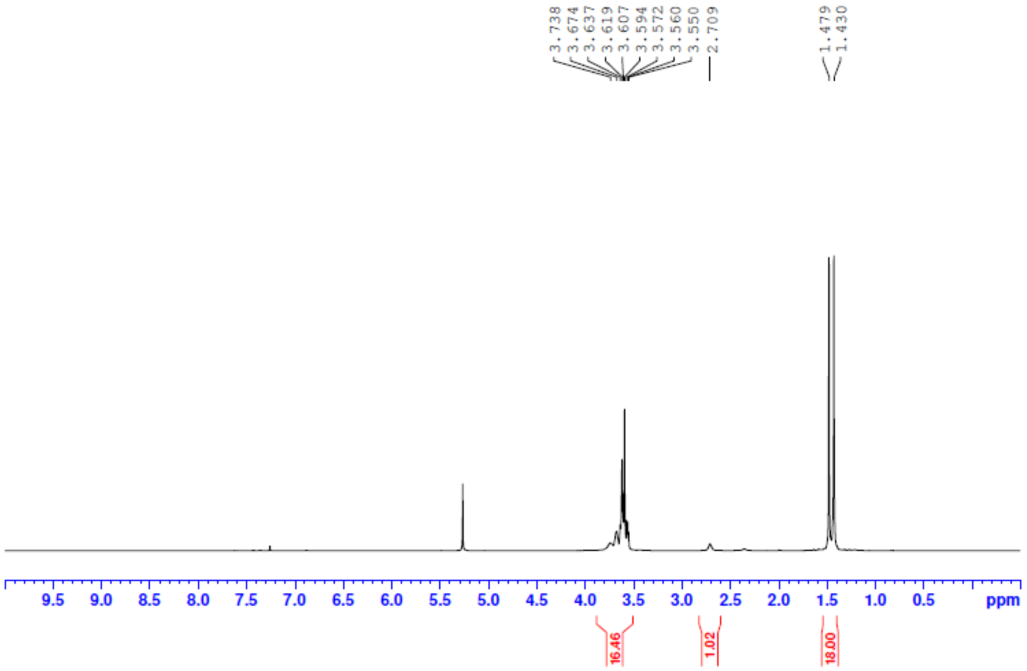

^{13}C-NMR data

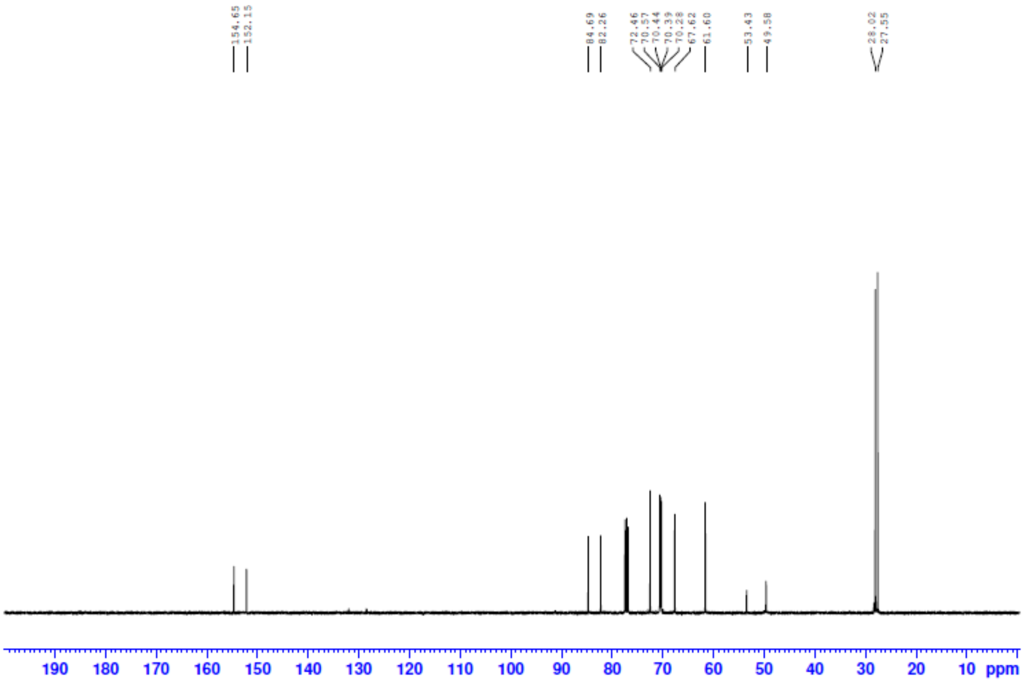

4.2. Compound 3

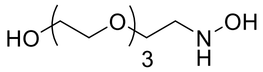

3

^{1}H-NMR data

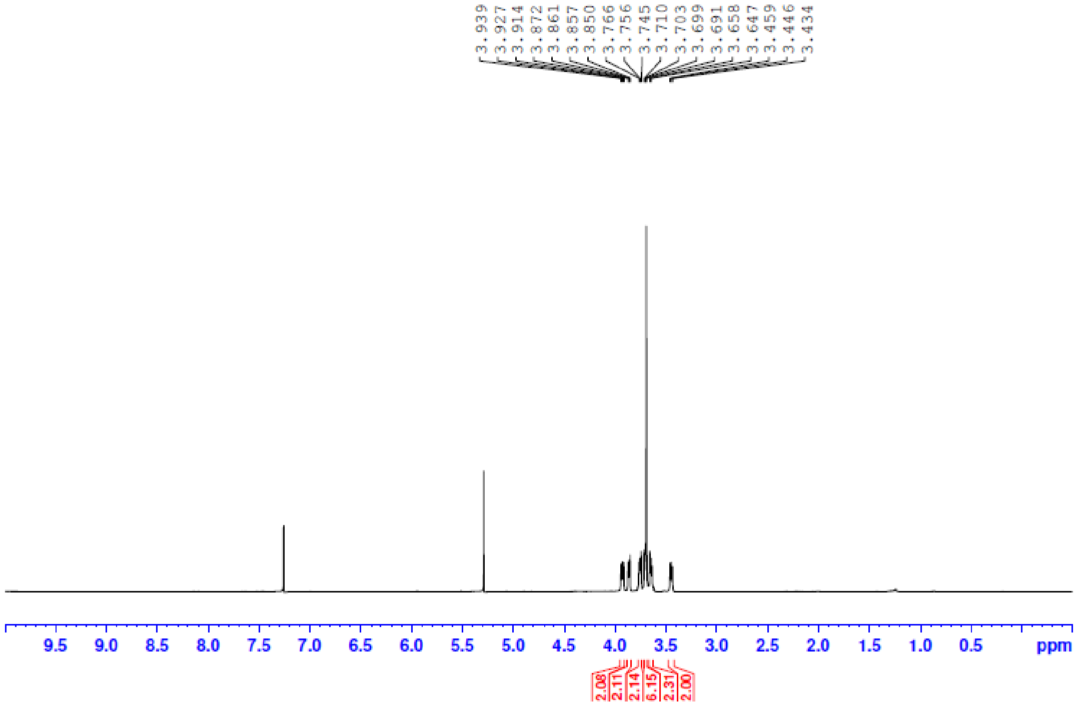

4.3. Probe 1

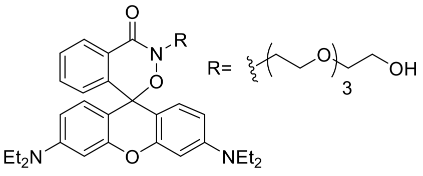

1

^{1}H-NMR data

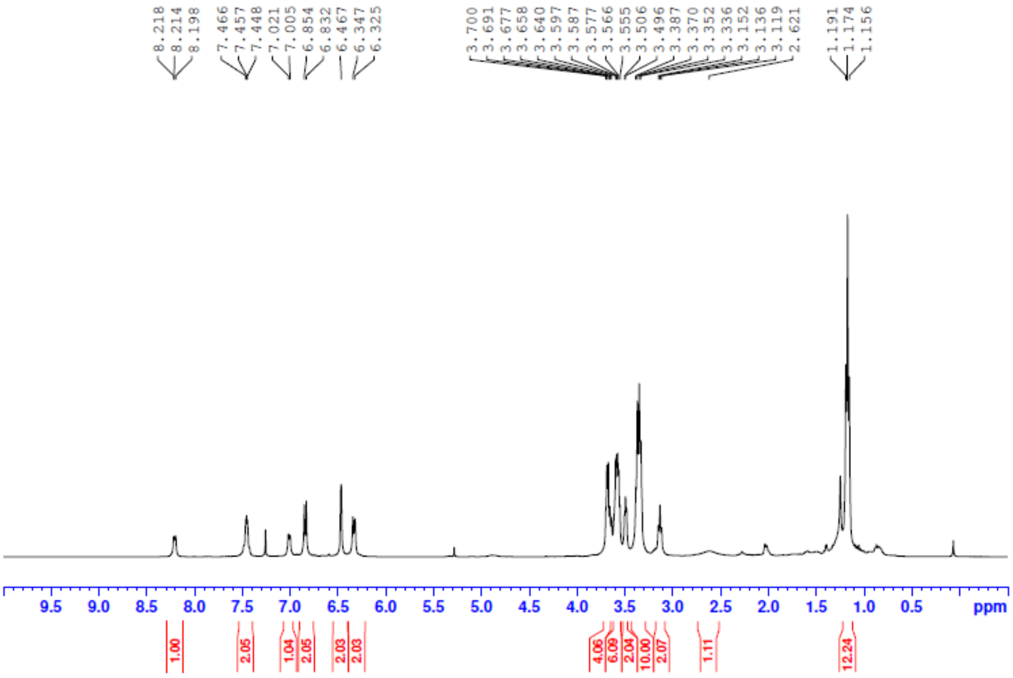

^{13}C-NMR data

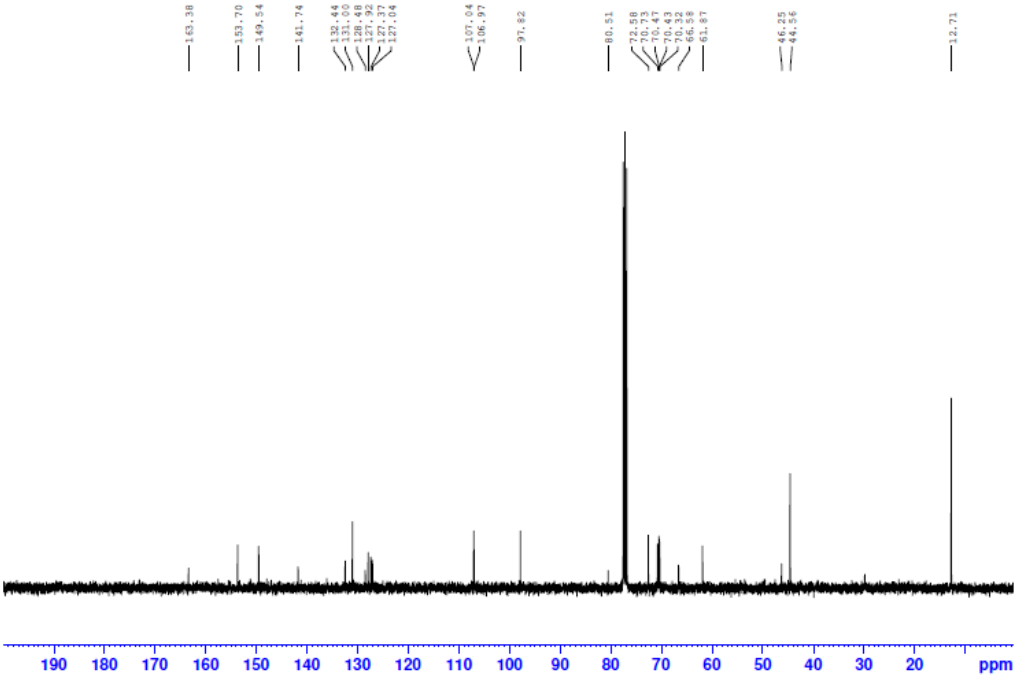

Supplement: Supplementary file 1 [file ijms-24-15073-s001.zip › ijms-2633797-supplementary.pdf]
